# Localized pleural metastasis without other organ metastases after nephrectomy for renal cell carcinoma

**DOI:** 10.1016/j.rmcr.2021.101388

**Published:** 2021-03-19

**Authors:** Zenya Saito, Kenichi Hata, Saiko Nishioka, Kentaro Tamura, Nobumasa Tamura, Masahiro Yoshida, Kentaro Kasa, Jun Hirano, Masataka Masubuchi, Kazuyoshi Kuwano

**Affiliations:** a Division of Respiratory Diseases, Department of Internal Medicine, Atsugi City Hospital, Kanagawa, Japan; b Department of Urology, Atsugi City Hospital, Kanagawa, Japan; c Department of Surgery, Atsugi City Hospital, Kanagawa, Japan; d Division of Thoracic Surgery, Department of Surgery, The Jikei University School of Medicine, Tokyo, Japan; e Division of Respiratory Diseases, Department of Internal Medicine, The Jikei University School of Medicine, Tokyo, Japan

**Keywords:** Renal cell carcinoma, Pleural effusion, Pleuritis, Metastasis

## Abstract

We present a case of a 69-year-old man who had localized pleural metastasis without other organ metastases after nephrectomy for right renal cell carcinoma (RCC). He complained of respiratory symptoms for more than two years after the operation and was confirmed to have right pleural effusion and multiple pleural masses on computed tomography (CT). There were no abnormal findings in the other organs, but the pleural mass gradually increased in size on CT. We suspected malignant tumors such as malignant pleural mesothelioma and synovial sarcoma in addition to RCC metastasis. Finally, we performed surgical resection of the pleural mass under general anesthesia, and we diagnosed pathologically as metastasis from RCC. Distant metastases of RCC are common in the lungs, bones, brain, and liver. To our knowledge, localized pleural metastases from RCC is rare.

## 1 Introduction

Renal cell carcinoma (RCC) most often metastasizes hematogenously to the lungs, and pleural metastases generally accompany lung metastases. Saito reported that 154 of 1451 (12%) autopsy cases of RCC had pleural metastases, but none of them had localized pleural metastasis [1]. In addition, Chernow et al. reported that only 1 of 96 patients with pleural metastasis had RCC as the primary cancer [2]. Here, we reported a rare case of RCC with localized metastatic masses in the pleura. The existence of the Batson venous plexus, which does not pass through the lung, has been revealed as the mechanism of metastasis to the pleura [3]. This plexus is a venous network that has no valve surrounding the spine; it extends from the skull to the pelvis, creating anastomoses with the odd veins, semi-odd veins, bronchial veins, and intercostal veins. It forms retrograde blood flow by changes in intrathoracic and intraabdominal pressures, causing tumor metastasis to the pleura. Since 2008, the therapeutic effects of molecular targeted drugs and immune checkpoint inhibitors have been observed for renal cancer. Particularly in cases of metastatic pleural tumors, in which only few can undergo highly invasive operation, longer prognosis can be expected with these drugs. Throughout this case report, we reviewed the diagnostic method and effective treatment, with reference to past similar cases.

## 2 Case presentation

**Fig. 1:**
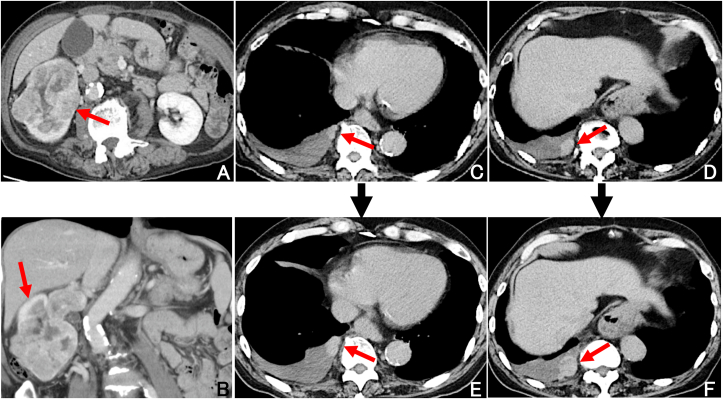
(A, B) Contrast-enhanced CT shows right RCC before nephrectomy. (C, D) Non-enhanced CT shows multiple right pleural tumors with pleural effusion. (E, F) Non-enhanced CT shows rapid growth of the pleural mass after one month.

**Fig. 2:**
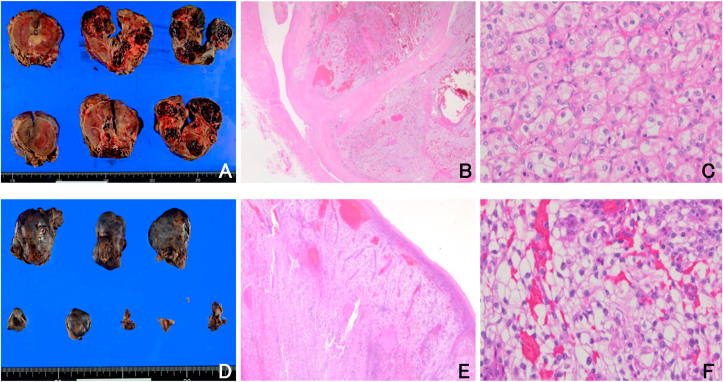
Histopathologic examinations of kidney and pleural specimens show primary and metastatic clear cell carcinoma. (A) Macroscopic image of the excised kidney tumor. (B): Hematoxylin–Eosin (H&E) staining of the kidney, × 40. (C): H&E staining of the kidney, × 200. (D) Macroscopic image of the excised pleural tumor. (E) H&E staining of the pleura, × 40. (F) H&E staining of the pleura, × 200.

**Fig. 3:**
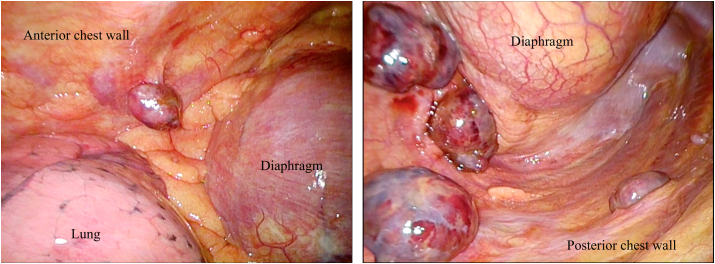
Thoracoscopic shows multiple hemorrhagic soft encapsulated tumors.

We encountered a 69-year-old man who had localized right pleural metastases after undergoing nephrectomy in September 2017 for right RCC (8.0 × 7.0 × 7.0 cm), clinical stage 3, clear cell type histology, grade 2 (Fig. 1, Fig. 2A–2C). The tumor was histologically solid, with hemorrhage, necrosis, scarring, and pseudocapsule formation, and infiltrated into the renal parenchyma. The tumor spread to the right renal vein, but no infiltration into the inferior vena cava was observed. He didn't receive adjuvant chemotherapy following the nephrectomy. Postoperatively, he did not present with any symptoms and received regular follow-up with chest and abdominal computed tomography (CT) every three months. There were no confirmed recurrence and metastasis until March 2020, when he visited our hospital complaining of right chest pain and dyspnea. At this time, CT scan revealed right pleural effusion and multiple pleural masses (Fig. 1C and D). There were no abnormal radiologic findings in the other organs. He had no smoking history, no past medical history, and no asbestos exposure. We suspected the following possibilities: metastasis from RCC, malignant pleural mesothelioma, and synovial sarcoma. For investigation and diagnosis, we performed two pleural effusion tests before surgery, but both cytological results were class I. After one month, there were no new abnormal radiologic findings, but we confirmed that the tumors obviously grew on CT (Fig. 1E and F). Finally, we performed intrathoracic tumor resection under general anesthesia and diagnosed histologically as pleural metastasis from clear cell type RCC (Fig. 2D–F). Thoracoscopy showed multiple reddish, soft, bleeding encapsulated tumors (Fig. 3). The pleural fluid cytology had no malignant cells. A total of eight bleeding tumors were completely resected, with the largest measuring 5.0 × 3.8 × 2.2 cm. This case is currently being treated for pembrolizumab plus axitinib, which has been reported to be superior to sunitinib in overall survival and progression-free survival [4]. This was a very rare case of localized metastatic pleural tumors from RCC diagnosed by thoracoscopic surgical resection.

## 3 Discussion

According to a previous systematic review, complete surgical resection is expected to improve the survival rate of patients with distant metastases [5]. However, surgical operation seems to be possible on only few cases of RCC with pleural metastasis, because it is highly invasive and includes lung resection. Table 1 shows a summary of 15 cases including this case of RCC with localized pleural metastasis. Most patients were diagnosed by thoracoscopic, ultrasound-guided, or CT-guided biopsy and were treated by interferon (IFN) or chemotherapy not surgical operation. Total pleural pneumonectomy was performed on only one case (case 3). As a diagnostic procedure, pleural fluid cytology was performed on some cases, but it was not enough for a definitive diagnosis. In fact, the positive rate of pleural fluid cytology for cancerous pleurisy has been said to be about 60% [6]. We suggest performing pleural biopsies, if possible, for definitive diagnosis.

**Table 1:** Cases of RCC with localized pleural metastasis.

Case	Age/Sex	Side		CT	Diagnostic procedure	Pathology	Operation		Additional therapy	Prognosis
		RCC	effusion				Nephrectomy	Pneumonectomy		
1 [10]	67/M	Left	Left	Pleural mass with pleural effusion	Autopsy	Metastatic renal cell carcinoma	Undone	Undone	BSC	Died after 4 months
2 [11]	71/M	Right	Left	Pleural thickening with pleural effusion	Autopsy	Metastatic renal clear cell carcinoma	Undone	Undone	BSC	Died after 2 months
3 [12]	50/M	Left	Left	Pleural thickening with pleural effusion	Thoracoscopic pleural biopsy	Adenocarcinoma and clear cell carcinoma	Done	Done	BSC	Survived after 15 months
4 [13]	66/M	Right	Left	Pleural mass with pleural effusion	Ultrasound-guided percutaneous biopsy	Metastatic renal clear cell carcinoma	Done	Undone	IFN-α	Improved
5 [14]	91/F	Left	Right	Pleural mass	Ultrasound-guided pleural biopsy	Metastatic renal cell carcinoma	Undone	Undone	Not referred	unknown
6 [15]	67/M	Right	Right	Pleural mass with pleural effusion	Video-assisted thoracoscopy	Metastatic renal cell carcinoma	Done	Undone	Chemotherapy (temsirolimus) for metastasis 3 years after nephrectomy	Survived after 10 months
7 [16]	69/M	Left	Left	Pleural mass	Fine-needle aspiration biopsy	Metastatic renal cell carcinoma	Done	Undone	IFN-α for metastasis 6 years after nephrectomy	Survived after 92 months
8 [17]	68/M	Left	Right	Pleural mass with pleural effusion	Under-assisted thoracoscopic biopsy	Metastatic renal clear cell carcinoma	Done	Undone	IFN-α and thoracic radiation for metastasis 16 years after nephrectomy	Died after 15 months
9 [18]	51/M	Left	Right	Pleural mass with pleural effusion	EBUS-TBNA	Metastatic papillary renal cell carcinoma	Not referred	Not referred	Not referred	Not referred
10 [19]	71/F	Left	Right	Pleural effusion	Thoracoscopic pleural biopsy	Metastatic renal cell carcinoma	Done	Undone	Chemotherapy (sunitinib) for metastasis 6 years after nephrectomy	Unknown
11 [20]	34/M	Left	Right	Pleural thickening with pleural effusion	CT-guided pleural tap	Metastatic renal cell carcinoma	Undone	Undone	High-dose IL-2 therapy, chemotherapy (tyrosine kinase inhibitor), radiation to chest wall	Unknown
12 [21]	61/M	Right	Left	Mass-like pleural thickening	Thoracoscopic pleural biopsy	Metastatic renal clear cell carcinoma	Done	Undone	Chemotherapy (sunitinib)	Improved after 6 months
13 [22]	75/M	Left	Right	Pleural mass with pleural effusion	Thoracoscopic pleural biopsy	Metastatic renal cell carcinoma	Done	Undone	Chemotherapy (sunitinib) for metastasis 1 year after nephrectomy	Mass reduction after 3 months
14 [23]	79/F	Left	Left	Mass-like pleural thickening	Thoracoscopic pleural biopsy	Metastatic renal cell carcinoma	Done	Undone	IFN	Survived after 5 months
This case	69/M	Right	Right	Pleural mass with pleural effusion	Thoracoscopic pleural biopsy	Metastatic renal clear cell carcinoma	Done	Undone	Chemotherapy (pembrolizumab plus axitinib)	Under treatment

BSC, best supportive care; CT, computed tomography; EBUS-TBNA, endobronchial ultrasound-guided transbronchial needle aspiration; IFN, interferon; IL, interleukin; RCC, renal cell carcinoma.

In general, RCC has a mean diameter growth rate of 0.59 cm/year and volume growth rate of 19.1 cm^3^/year, and tumors <4 cm show even lower growth rates [7]. However, compared with primary tumors, which have very slow growth, metastatic pleural mass may progress rapidly, as in this case. In fact, in this case, the metastatic pleural mass obviously grew on CT after one month (Fig. 1B–E). As shown in Table 1, some cases developed recurrence after more than five years of nephrectomy, consistent with the feature of slow growth. Moreover, case 8 had metastatic pleural tumors 16 years after nephrectomy and died 15 months later, despite treatment. Therefore, we need to pay attention to the appearance of respiratory symptoms and pleural effusion, and longer follow-up may be required even after nephrectomy.

With regard to treatment, IFN and interleukin (IL) have been used for advanced RCC, but their reported response rates have been low at 5%–27% [8,9]. At present, the number of treatment drugs has increased with the advent of molecular targeted and immunotherapy. The effects of these drugs are highly expected, especially in patients with pleural metastases, among whom only few can undergo surgery. In fact, reduction in the size of the pleural mass was confirmed in some cases (cases 4, 12, and 13). In the future, more similar case reports are required to determine the better treatment in patients with pleural metastases from RCC.

## 4 Conclusions

Localized metastatic pleural mass secondary to RCC is extremely rare, and most cases have been treated by IFN, IL, and chemotherapy not surgery. More information about the therapeutic effects and prognosis of cases of pleural metastases from RCC needs to be collected.

## Funding

This research received no specific grant from any funding agency in the public commercial, or not-for-profit sectors.

## Ethical approval

Informed consent was obtained from the patient and it is available upon request.

## Author contribution

Z.S. designed the study and drafted the manuscript. K.H., S.N., K.T., N.T., M.Y., K.K., J.H., M.M., K.K. contributed to review of this manuscript. All authors read and approved the final manuscript.

## Declaration of competing interest

The authors declare that they have no known competing financial interests or personal relationships that could have appeared to influence the work reported in this paper.
